# New version of the emotion socialization scale with the positive emotion of overjoy: initial validation evidence with Portuguese adolescents

**DOI:** 10.1186/s41155-018-0090-3

**Published:** 2018-04-03

**Authors:** Eva Costa Martins, Fernando Ferreira-Santos, Liliana Meira

**Affiliations:** 1 Department of Social and Behavioural Sciences, Maia University Institute–ISMAI/CPUP, Av. Carlos Oliveira Campos, 4475-690 Maia, Portugal; 2 Laboratory of Neuropsychophysiology, Faculty of Psychology and Education Sciences, University of Porto, Porto, Portugal

**Keywords:** Instrument validation, Positive emotion, Parental emotion socialization strategies’ scale, Maternal rearing practices, Adolescence, Confirmatory factor analysis

## Abstract

There are few studies on parental socialization of positive emotions in adolescents and few instruments that measure these parental reactions. Therefore, we developed a new version of the Emotion Socialization Scale (ESS) for the positive emotion of overjoy. We further provided some evidence of validity and reliability of the Portuguese ESS, featuring overjoy, fear, anger, and sadness. Adolescents (*N* = 418) answered questionnaires on maternal emotion socialization and maternal rearing practices. Confirmatory factor analysis achieved good (*reward*, *neglect*, *override*, *magnify*) to acceptable (*punish*) levels of fit, and scales had good levels of internal consistency, except for punish (all emotions) and neglect (overjoy). Association with maternal rearing practices supported the adaptive role of reward and magnify and the less adaptive role of punish, override, neglect of positive emotion, with some exceptions. This investigation demonstrated the importance of assessing parents’ reactions to adolescents’ positive emotion as these may be important indicators of the parent-adolescent relationship quality.

**Electronic supplementary material:**

The online version of this article (10.1186/s41155-018-0090-3) contains supplementary material, which is available to authorized users.

## Background

Parental emotion socialization encompasses processes that impact the development of youth’ emotion-related skills―emotion understanding, expression, and regulation―through parent-child exchanges (Eisenberg, Cumberland, & Spinrad, 1998). Direct emotion socialization features parental reactions to their youth’s expression of emotion, how they discuss emotions with their children, and how parents coach emotion regulatory efforts (Eisenberg et al. 1998). The relevance of parental emotion socialization practices to positive developmental and to youth psychopathology has been highlighted (Katz et al. 2014). However, most research has been conducted on negative emotions (NE). This article aims at broadening this scope by developing an instrument that measures parental socialization practices of overjoy/excitement.

Two socialization styles have been described: emotion coaching vs. emotion dismissing (Lemerise, 2016). Emotion coaching is associated with broader psychological and peer adjustment and describes parents that tend to be aware of their children’s emotions, to accept their emotional expression and supportive of their regulation by helping youth learn appropriate emotion regulatory strategies. In the contrary, emotion dismissing is associated with less adaptive trajectories. These parents are less aware of their youth emotional life, show unacceptance to their emotion expression, with dismissing, disapproving, and punitive reactions. In consequence, these reactions do not support the development of child’s emotion regulatory capacities.

Based on Tomkins (1963) work, (Magai, C.: Emotions as a child. Unpublished manuscript.) proposed the Emotions as a Child Scales Inventory (EAC) that includes the Emotion Socialization Scale (ESS). The ESS assesses youth’s perception about how parents react to their NE and features five parental emotion socialization strategies (Klimes-Dougan et al., 2007; O’Neal & Magai, 2005). The positive strategy, *reward,* comprises parental behaviors that foster the reduction of negative affect, while promoting the adolescent’s tolerance to all NE by providing comfort, empathizing, and problem solving (Malatesta-Magai, 1991). In contrast, the four remaining strategies do not promote the reduction of NE and the capacity to tolerate and regulate these emotions (Malatesta-Magai, 1991). *Punish* encompasses disapproval for the emotion expressed by the adolescent or making fun of the adolescent. *Neglect* involves behaviors that ignore the youth’s emotion and marks parental unavailability to the adolescent when expressing that emotion. *Override* covers parental behaviors that have the objective of silencing/downplaying the expression of the emotion, namely, dismissive (telling a child expressing sadness to cheer up) or distracting behaviors (giving a present to a sad adolescent). Finally, *magnify* entails parents expressing the same emotion with equal or stronger intensity. Several versions of the ESS have been developed for youth, caregivers, peers, and adults as respondents (Kehoe, Havighurst, & Harley, 2014; Klimes-Dougan et al. 2014; Magai, Consedine, Gillespie, O’Neal, & Vilker, 2004; Sanders, Zeman, Poon, & Miller, 2015).

Overall, Tomkins and Magai’s predictions have been supported (Kehoe et al. 2014; Klimes-Dougan et al. 2007; O’Neal & Magai, 2005), with contradictory findings regarding override, as it is not always associated with negative outcomes (Klimes-Dougan et al. 2007, 2014). Override also includes distracting parental behaviors that in conjunction with an emotionally acceptant stance may be supportive of youths’ regulatory efforts (Klimes-Dougan et al. 2014). These authors also question the negative role of magnify, as in certain circumstances, these may be interpreted by youth as empathic responding. More importantly, O’Neal and Magai (2005) showed that the adaptive quality of each socialization strategy depends on the emotion (e.g., sadness vs. anger) and the outcome (internalization vs. externalization) under study.

Even in studies using other instruments beside ESS, only a handful have directed their attention to socialization practices regarding positive emotion (PE; Bai, Repetti, & Sperling, 2016; Gentzler, Ramsey, & Black, 2015; Katz et al. 2014; Yap, Allen, & Ladouceur, 2008). Thus, since this is a largely unexplored topic in the literature, we analyzed the factorial structure of the socialization practices regarding the positive emotional state of *overjoy (excited)* contained in an unpublished version of the ESS (Magai, C., & O’Neal, C. R.: Emotions as a child (child version). Unpublished manuscript.) to obtain some evidence of validity (Urbina, 2014). This task was completed after adapting the overjoy items following recent theoretical developments on PE. Given that pleasant emotions are typically rated with lower levels of arousal than unpleasant emotions (Almeida et al. 2016; Soares et al. 2015), overjoy is more appropriate than joy to match the emotional intensity of the other NE of the ESS.

PE are associated with several beneficial outcomes in the personal and social realms: increased cognitive flexibility, motivation, social connectedness, health, resilience, emotion regulation, improved coping with grief, and in adolescents with reward-seeking behavior (Fredrickson, 2013; Gruber, Devlin, & Moskowitz, 2014). Therefore, it is desirable that parents foster the expression of PE, in contrast with socialization practices for NE that should promote the mitigation of such emotions (Bai et al. 2016; Gentzler et al. 2015). Recent results seem to support this theoretical view by linking attachment insecurity and psychopathology to socialization practices that diminish PE expressions in youth (Gentzler et al. 2015; Katz et al. 2014; Yap et al. 2008).

Like with NE, rewarding PE can be considered an adaptive socialization strategy, while punishing and neglecting them cannot. Override is expected to be maladaptive as well as it tends to diminish PE. On the contrary, magnify should be considered a positive strategy. When parents join adolescents in their positive emotional expression, they reinforce the expression of that emotion in the adolescent, as expressing PE signals agreement with what others are doing (Clark & Monin, 2014). Also, by sharing PE with their youth, they also intensify the original pleasurable experience (Bai et al. 2016). In summary, magnify fosters *capitalizing*, an adaptive emotion regulation strategy, that augments or prolongs PE through social-contact-seeking behaviors such as communicating, sharing, or celebrating with others (Gentzler, Morey, Palmer, & Yi, 2013; Langston, 1994). It also fosters *savoring*, that is, the capacity to draw pleasure from positive experiences through anticipation, present enjoyment, and reminiscence (Bryant, 2003; Gentzler et al. 2013).

The first objective of this study was to adapt the emotion socialization strategies assessed by the ESS to the positive emotional state of overjoy based on the theoretical framework presented in this article. ESS is frequently used for the study of socialization practices, and there is no similar instrument in Portuguese, a language spoken by more than 261 million people all over the world, according to the Portuguese Language Observatory (2015). Moreover, the questionnaire’s format is coherent with previous research showing that emotional socialization practices may be emotion-specific (O’Neal & Magai, 2005), so the ESS included four emotions: sadness, anger, fear, and overjoy. Another distinctive feature of EES is that it assesses youth’s perception of their parents’ general response pattern, rather than parents’ responses to specific emotional scenarios or as parent reports, as other available instruments (Gentzler et al. 2015; Yap et al. 2008).

The second objective was to translate the ESS (Magai & O’Neal, 1997) and to provide some evidence of the questionnaire’s scores inference validity, through the study of the questionnaires’ factor structure and convergent validity (by relating ESS with maternal rearing practices (MRP)). We also investigated the instruments’ scores reliability. We studied the factor structure, through confirmatory factor analysis (CFA). We aimed to confirm that the questionnaire’s internal structure depicts the five parental emotion socialization strategies hypothesized to exist by, even in the case of the new positive emotion of overjoy. CFA is indeed an analysis frequently used as evidence of validity because it assesses if the item co-variation can be explained by the construct’s theoretical structure, that is, the latent trait model (Baghaei & Yazdi, 2016).

Additionally, MRP were used to assess one evidence of convergent validity, based on the assumption that parental behaviors in response to youth’s emotion are related with maternal practices, attitudes, and beliefs about parenting (Katz, Wilson, & Gottman, 1999). For the sake of simplicity, we used adolescents’ reports on MRP, as prior research has shown that in mother-father dyads, each partners’ responses are predicted by the other element of the couple (Nelson et al. 2016). MRP refer to the overall strategies used by parents to socialize their child, namely, through warmth and discipline (Baker & Hoerger, 2012). Findings suggest that there are three main dimensions of parental rearing behaviors: emotional warmth, associated with positive developmental outcomes, and overprotection and rejection, associated with negative outcomes (Baker & Hoerger, 2012).

Given the above, we hypothesized that, in response to PE, magnify should be an adaptive emotion socialization strategy, and override a maladaptive strategy. We therefore expected that reward (PE and NE), magnify (PE), and override (NE) should correlate positively with emotional warmth and negatively with rejection or overprotection. The inverse relation was expected for punish, neglect (PE and NE), override (PE), and magnify (NE).

## Methods

### Participants

Participants were 418 Portuguese adolescents (57.7% girls), ranging from 13 to 19 years old (*M* = 14.75, SD = 1.31), attending the seventh (2.4%), eighth (54.8%), ninth (20.6%), tenth (7.2%), eleventh (11%), and twelfth (4.1%) grades. Most mothers had 6 to 9 years of education (48.6%) followed by 4 years (22.7%), 12 years (17.9%), and university level education (9.3%). Participants were recruited in six elementary and high schools located at four northern Portuguese cities by convenience sampling, after obtaining the permission from the *Direção-Geral da Educação* (managing body of the Portuguese Education System) and individual school administrations. A research assistant with the help of the form teacher presented the study to the adolescents. Parents were sent home informed consents, and the adolescents that had their parents’ signed document were included in the study. Adolescents signaled by the form teachers and the school services with cognitive impairment were excluded from data collection, as this would impair them to fill-out the questionnaires independently. We aimed for a sample size of around 400 participants as it is considered adequate for CFA of 4 to 6 factors with 3 items per factor (assuming modest loadings of .40 for all variables; Jackson, Voth, & Frey, 2013).

### Procedures

The questionnaires that are featured in this article were part of a larger assessment protocol regarding adolescents overall functioning and skills (e.g., social problem-solving skills and social support). Questionnaires were administered in a classroom setting under the supervision of the assistant researcher, and anonymity of the data was ensured. Instructions given to the adolescents included the presentation of the study’s goals (i.e., to assess several variables related to adolescent’s overall functioning), the presentation of the protocol, and the request to complete all the items of each questionnaire. After this initial presentation, questionnaires were distributed to participants, who filled them out autonomously. The assistant researcher did not read the questionnaires out loud to the participants and did not look for missing answers when the adolescent delivered the questionnaires.

### Instruments

#### Emotion socialization strategies

The ESS (Magai, C., & O’Neal, C. R.: Emotions as a child (child version). Unpublished manuscript.) youth self-report assessed five parental socialization strategies in questionnaire format (more detailed description in the introduction): reward; punish; neglect; override; and magnify. The five parental socialization strategies were assessed separately for each emotion, totaling 20 scales. Adolescents were asked to rate the frequency of their parents’ reaction (60 items) when they expressed a particular emotion―sadness, anger, fear, and overjoy―(15 items per emotion) on a 5-point Likert scale (1 = never, 3 = sometimes, 5 = very often). The questionnaire structure and item formulation were the same for each emotion (see Table 1 for sample items).

**Table 1:** Emotion socialization strategies for overjoy: item scale composition and changes conducted for the present study

Original version	Changes conducted
Reward	
3. When I was overjoyed, my parent/caregiver helped me deal with the issue that made me overjoyed.	3. When I was overjoyed, my parent/caregiver helped me appreciate the reasons that made me overjoyed.
6. When I was overjoyed, my parent/caregiver asked me what made me overjoyed.	
15. When I was overjoyed, my parent/caregiver comforted me.	15. When I was overjoyed, my parent/caregiver shared my happiness.
Magnify	
4. When I was overjoyed, my parent/caregiver got *very* overjoyed.	
8. When I was overjoyed, my parent/caregiver expressed that s/he was *very* overjoyed.	
13. When I was overjoyed, my parent/caregiver got *very* upset.	13. When I was overjoyed, my parent/caregiver got *very* excited.
Punish	
2. When I was overjoyed, my parent/caregiver told me to stop being overjoyed.	
5. When I was overjoyed, my parent/caregiver told me that I was acting younger than my age.	
9. When I was overjoyed, my parent/caregiver let me know s/he did not approve of my being overjoyed.	
Neglect	
1. When I was overjoyed or overexcited, my parent/caregiver responded to my being overjoyed or overexcited.	
12. When I was overjoyed, my parent/caregiver took time to focus on me.	
14. When I was overjoyed, my parent/caregiver did not pay attention to my being overjoyed.	
Override	
7. When I was overjoyed, my parent/caregiver told me not to worry.	7. When I was overjoyed, my parent/caregiver told me to worry about other stuff.
10. When I was overjoyed, my parent/caregiver bought me something I liked.	
11. When I was overjoyed, my parent/caregiver told me to cheer up.	11. When I was overjoyed, my parent/caregiver told me to calm down.

*Note.* Empty spaces indicate that there were no item changes

##### Adaptation for overjoy

One of the original authors, Colleen R. O’Neal, provided us with an unpublished version of the ESS that included overjoy, but with the exact structure and phrasing of the other NE. Therefore, we conducted changes in the wording of five items (Table 1). Colleen R. O’Neal agreed with our changes. The modifications were in line with the following arguments. Parenting behaviors that promote adolescents’ PE seems to be adaptive (Gentzler et al. 2015; Katz et al. 2014; Yap et al. 2008). Therefore, we expected reward and magnify to be adaptive emotion socialization strategies, because they upregulate PE, while punish, neglect, and override would not, because they downregulate them.

In the reward and magnify scales, item 13 was altered from “got very upset” to “got very excited” and item 15 from “comforted me” to “shared my happiness” because when parents express the same PE as their adolescents, they support that adolescents’ capitalize on the positive experience. Regarding item 3, we substituted “deal with the issue” by “appreciate the reasons” because this parental response fosters adolescents savoring PE. In override item, 7 we substituted “not to worry” with “to worry about other stuff” (not appreciating the positive experience leading to its early demise) and in item 11, we changed “told me to cheer up” to “to calm down” (parents are explicitly saying to downregulate the emotion).

##### Translation

The ESS was developed using the English version ((Magai, C., & O’Neal, C. R.: Emotions as a child (child version). Unpublished manuscript.) through the back-translation method (Hambleton, 2005). The original version was translated into Portuguese and questions that arouse were discussed with Colleen R. O’Neal. The Portuguese version was, then, back-translated into English by another speaker fluent in both languages. Next, the original version was compared with the back-translation and discrepancies were solved by consensus. A test of the pre-final version was conducted with two adolescents, which included an interview to determine comprehension of each question. No relevant adjustments were made to the final version of the questionnaire.

#### Maternal rearing practices

The Parental Rearing Style Questionnaire for Use with Adolescents: EMBU-A (Gerlsma, Arrindell, van der Veen, & Emmelkamp, 1991) was used to assess the adolescents’ perception of three MRP. *Emotional warmth* refers to positive MRP that include parental support and nurturing, positive reinforcement, supervision, active involvement in child’s life, and constructive discipline. *Overprotection,* encompasses negative MRP that include excessive control of child’s activities, excessive contact and support, showing high expectations related the child’s performance, and inflexibility towards behavioral and routine rules. Finally, *rejection,* another negative practice, is characterized by hostility and neglect, rigid discipline, obedience demanding, physical and verbal punishment. For the Portuguese version used in this study (48 items; each with a 4-point Likert scales), validity evidence has been provided for the original three-scale structure through a principal component analysis with a Portuguese adolescent sample (Lacerda, M.: Percepção das práticas parentais pelos adolescentes: Implicações na percepção do controlo e nas estratégias de coping, Unpublished master’s thesis.). The internal consistency for each mother scale was as follows: emotional warmth, α = .91, overprotection, α = .64, and rejection, α = .87. In our study, we used the mothers’ scales, which showed good internal consistency: emotional warmth, α = .91; overprotection, α = .71; and rejection, α = .85.

### Data analysis

We began by conducting a missing data analysis. The highest percentage of missing values for EMBU-A was 0.7% on item 31 and for the ESS was 1.2% for items 11 (anger); 5, 6, and 8 (fear). The maximum number of blank items in a single questionnaire was 33 items in EMBU-A and 29 items in ESS. Considering the low percentage of missing values for EMBU-A and ESS (< 10%), the IBM SPSS 23 imputation regression method can be considered adequate (Manly & Wells, 2015), so we used it for missing data imputation.

**Fig. 1:**
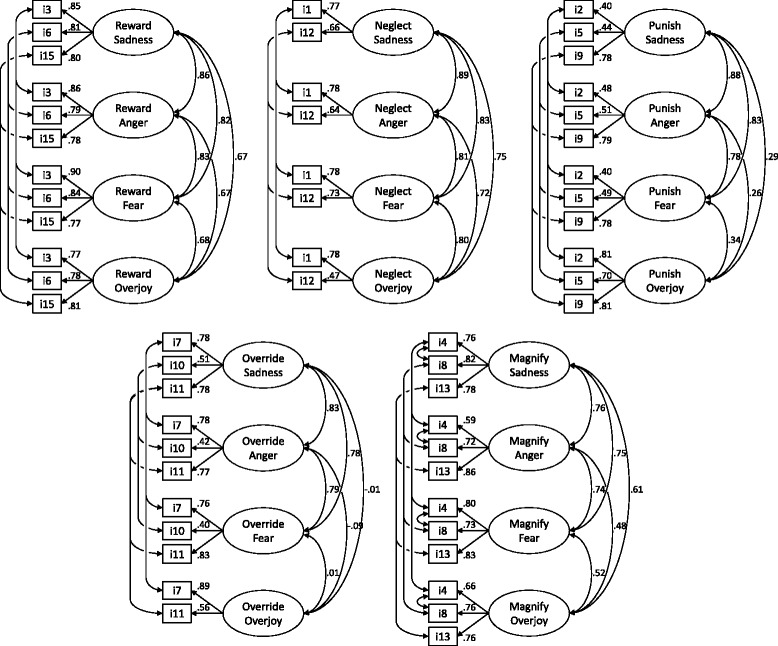
Path diagrams for the final five models of emotion socialization strategies. *i* = item. For simplicity, item errors are omitted

After the final dataset was complete, we started by testing if the questionnaire’s internal structure depicted the five parental emotion socialization strategies hypothesized to exist by, even in the case of the new positive emotion of overjoy. The factorial structure of the ESS was studied by conducting confirmatory factor analysis (CFA) using AMOS 20. Five CFA were carried out (one for each socialization strategy, e.g., reward; see Fig. 1) using maximum likelihood estimation. So in each model, we assessed if the socialization strategy (e.g., reward) could be identified in all four emotions (anger, fear, sadness, and overjoy), maintaining the original ESS structure: three items per socialization scale. Correlations between residuals of the items with parallel phrasing were allowed across emotions (e.g., item 1 in sad, anger, fear, and overjoy) as it has been done in CFA of the English versions of the ESS. Standardized factor loadings were inspected so that items not contributing significantly to the factors were excluded. Finally, all models’ fit was assessed by comparing several indicators of fit (Hooper, Coughlan, & Mullen, 2008): CFI = comparative fit index (good fit > .95); TLI = Tucker–Lewis index (good fit > .95); RMSEA = root-mean-square error of approximation (good fit < .07); SRMR = Standardized Root Mean Square Residual (good fit < .08). We, then, analyzed the internal consistency of each emotion socialization strategy by emotion with Cronbach’s alpha (20 scales).

Finally, descriptive statistics for the study variables were presented followed by Pearson correlations between the emotion socialization scales and the MRP. Pearson statistic aimed to look for an evidence of the questionnaires’ convergent validity.

## Results

### Factorial structure and internal consistency

The factorial structure of the ESS was studied by conducting confirmatory factor analysis (CFA) using AMOS 20. Five CFA were carried out, using maximum likelihood estimation, in order to test for the expected five emotions socialization strategies using the original ESS structure: three items per scale (Additional file 1). Standardized factor loadings were inspected and items with non-significant loadings were excluded: item 14 (in all emotions) and item 10 (in overjoy). The five final models are presented in Fig. 1. Correlations between residuals of the items with parallel phrasing were allowed across emotions (e.g., item 1 in sad, anger, fear, and overjoy). Also, after inspection of modification indices, in the magnify model, we decided to correlate residuals of item 4 (*got very angry*) and item 8 (*was very angry*), within the same emotion, because they also overlap on the wording used.

Model fit indices are reported in Table 2. Good levels of fit were found for the models of all emotion socialization strategies, with the exception of punish that reached acceptable levels of fit. Standardized factor loadings for reward items ranged from .90 (fear_3) to .77 (fear_15), *M*_loading_ = .81; for punish, ranged from .81 (overjoy_9) to .40 (sadness_2), *M*_loading_ = .62; for neglect varied from .79 (fear_1) to .47 (overjoy_12), *M*_loading_ = of .70; for override ranged from .87 (overjoy_7) to .41 (fear_10), *M*_loading_ = .69; and finally for magnify, standardized loadings varied from .86 (anger_13) to .59 (anger_4), *M*_loading_ = .76.

**Table 2:** Fit Indices for the five models of emotion socialization strategies (*N* = 418)

Model	*df*	*Χ* ^*2*^	CFI	TLI	RMSEA	SRMR
Reward	34	60.52**	.993	.987	.043	.034
Punish	34	113.10***	.962	.925	.075	.084
Neglect	6	11.35	.997	.985	.046	.020
Override	27	78.23***	.976	.952	.067	.069
Magnify	30	61.30**	.988	.973	.050	.038

Note. *CFI* = comparative fit index (good fit > .95), *TLI* = Tucker–Lewis index (good fit > .95), RMSEA = root-mean-square error of approximation (good fit < .07); SRMR = Standardized Root Mean Square Residual (good fit < .08)

*p < .05. **p < .01. ***p < .001

We analyzed the internal consistency of each emotion socialization strategy with Cronbach’s alpha (Table 3, see also for descriptives). Values reached acceptable to good internal consistency (16 out of 20 scales) with the following exceptions: punish (sad, anger, fear) and neglect (overjoy), .51 ≥ α ≤ .57.

**Table 3:** Descriptive statistics and Cronbach’s α for emotion socialization strategies and maternal rearing practices (*N* = 418)

	Min	Max	Range	M	SD	Cronbach’s α
Emotion socialization strategies						
Reward						
Sadness	1	5	4	3.57	1.19	.89
Anger	1	5	4	3.36	1.16	.87
Fear	1	5	4	3.45	1.23	.89
Overjoy	1	5	4	3.46	1.08	.83
Punish						
Sadness	1	5	4	2.67	.90	.51
Anger	1	5	4	2.59	.90	.57
Fear	1	5	4	2.45	.89	.53
Overjoy	1	5	4	1.69	.96	.80
Neglect						
Sadness	1	5	4	2.70	1.14	.68
Anger	1	5	4	2.96	1.09	.64
Fear	1	5	4	2.97	1.19	.76
Overjoy	1	5	4	2.81	1.04	.52
Override						
Sadness	1	5	4	3.18	.99	.72
Anger	1	5	4	2.90	.97	.68
Fear	1	5	4	2.98	.98	.67
Overjoy	1	5	4	2.15	1.02	.70
Magnify						
Sadness	1	5	4	2.48	1.07	.84
Anger	1	5	4	2.12	.97	.81
Fear	1	5	4	1.91	.94	.83
Overjoy	1	5	4	3.16	1.07	.80
Parenting practices						
Emotional warmth	1.15	4.00	2.85	3.26	.53	.90
Overprotection	1.18	3.64	2.45	2.07	.49	.71
Rejection	1.12	3.82	2.71	1.76	.48	.85

### Convergent validity

Correlations between emotion socialization strategies and MRP are displayed in Table 4. Regarding overjoy, our expectation was that that reward and magnify would be adaptive socialization strategies. This hypothesis was largely confirmed since both correlated positively with emotional warmth (adaptive MRP) and negatively with rejection (negative MRP). Non-significant relations were found with overprotection. Again, for overjoy, we hypothesized that punish, neglect, and override would be maladaptive emotion socialization strategies as they tend to downregulate PE. We indeed found that youth that perceived their mothers as using more of these strategies were also perceived as being more rejecting, more overprotective (non-significant relation with neglect), and less emotionally warm (non-significant relation with override).

**Table 4:** Correlations between emotion socialization strategies and sociodemographics and parenting practices (*N* = 418)

Emotion socialization strategies	Sociodemographics				Parenting rearing practices		
	Adolescents			Mothers			
	Sex^a^	Age	Years of education^a^	Years of education^a^	Emotional warmth	Overprotection	Rejection
Reward							
Sadness	.10*	− .03	−.01	.09†	.50***	− .12*	− .30***
Anger	.11*	− .03	−.02	.11*	.49***	− .10*	− .29***
Fear	.17***	− .04	−.02	.08	.45***	− .13**	− 33***
Overjoy	.19***	.01	−.03	.02	.40***	− .04	− .26***
Punish							
Sadness	.00	− .08†	−.07	− .04	.27***	.10*	− .02
Anger	− .02	− .03	−.04	− .01	.23***	.15**	.02
Fear	− .03	− .07	−.13**	− .05	.23***	.09†	− .01
Overjoy	− .20***	.01	−.14**	− .06	− .14**	.15**	.21***
Neglect							
Sadness	− .09†	.06	− .02	− .10*	− .45***	.14**	.30***
Anger	− .10*	.04	− .02	− .13*	− .40***	.04	.19***
Fear	− .16**	.03	− .05	− .11*	− .40***	.11*	.32***
Overjoy	− .14**	.01	.00	− .02	− .38***	.07	.29***
Override							
Sadness	.14**	− .08	− .12*	.01	.39***	− .08	− .25***
Anger	.06	− .07	− .12*	.06	.40***	− .07	− .21***
Fear	.09†	− .07	− .12*	.04	.35***	− .05	− .22***
Overjoy	− .07	− .04	− .09†	− .09†	− .06	.21***	.18***
Magnify							
Sadness	.02	− .07	− .16**	− .03	.35***	.10*	− .04
Anger	− .03	− .03	− .08	− .03	.11*	.20***	.14**
Fear	.01	− .02	− .16**	− .04	.12*	.18***	.06
Overjoy	.09†	.01	− .04	− .03	.37***	− .02	− .18***

^a^All correlations are Pearson correlations with the exception of the Point-Biserial correlations with sex and Spearman correlations with years of education

^†^*p* < .10, *p < .05, **p < .01, ***p < .001

For the NE, we had different expectations. We considered that reward and override were adaptive emotion socialization practices, while neglect, punish, and magnify were not. Indeed, parents perceived as using more reward and override were also more emotionally warm (positive MRP) and used less negative MRP, like rejection and overprotection (this last only for reward). Neglect use was associated with higher rejection and neglect of sadness and fear with overprotection; punish of sadness and anger with overprotection; and magnify with overprotection and magnify of anger with rejection. Finally, neglect was associated with less emotionally warm parenting, but contrary to our expectations, we found positive correlations with punish and magnify.

## Discussion

In this investigation, we provided some evidence of validity and reliability of the Portuguese version of the ESS and developed a version for the PE of overjoy, considering magnify as an adaptive strategy, and override as a maladaptive strategy in response to PE.

Our findings add to evidence regarding the usefulness and validity of the EES. The models for each emotion socialization strategy had good to acceptable levels of fit, and included minor changes in item composition: item 14 was excluded from neglect (all emotions), and item 10 from override (overjoy). Moreover, these models include the new positive emotional state of overjoy, and the changes in items made for the purpose of this study. Additionally, this questionnaire is comprised of five emotion socialization scales (e.g., reward) for each of the four emotions. Most emotion socialization scales scores (e.g., reward of sadness, reward of overjoy) achieved acceptable to good levels of internal consistency, 16 from a total of 20. This is a relevant result due to the high number of scales in the ESS and the small item composition. Finally, regarding some evidence of convergent validity, as expected, many of the purported adaptive emotion socialization strategies were associated with emotional warmth (positive MRP) and negatively with overprotection or rejection (both negative ERP), while the inverse relation was found for maladaptive emotion socialization strategies. However, some contrary findings also emerged for punish and magnify (NE). These will be examined further into the discussion.

PE are associated with several positive outcomes (Fredrickson, 2013; Gruber et al. 2014). We based our research on this framework, so that parental reactions to adolescents’ overjoy that validate and increase PE were considered adaptive. As predicted, we showed that parents perceived by the adolescent as using more supportive reactions to their expression of PE (reward) and of expressing the same emotion with equal or stronger intensity (magnify), were also viewed as being more emotionally warm/using constructive discipline (MRP: emotional warmth), and using less harsh parenting (MRP: rejection). It is possible that parents that use reward and magnify are helping their adolescent to capitalize on their positive emotional experience, because sharing PE with others increases and prolongs that experience (Gentzler et al. 2013; Langston, 1994). Also, by doing so, parents are signaling that experiencing PE is a “good thing.” This will foster adolescents’ ability to take the best of positive experiences, that is, to savor them (Bryant, 2003; Gentzler et al. 2013). Indeed, it has been demonstrated that adolescents that reported more celebrating, sharing, or reflecting on positive events and feelings report more positive affect (Gentzler et al. 2013). In conclusion, parental strategies that nurture youth’s positive emotional experiences may be considered adaptive, since in turn, PE have often been associated with flexibility and resilience (Fredrickson, 2013; Gruber et al. 2014). In contrast, parental reactions that buffer adolescents’ positive emotions, like override, punish, and neglect should be considered maladaptive strategies.

Our results give some support to the hypotheses that override, punish, and neglect are maladaptive emotion socialization strategies of PE. We theorized that they may be less optimal maternal socialization strategies for adolescent’s PE as they may lead the adolescent to experience fewer PE and/or less intense or durable. Indeed, we found that adolescents that perceived parents as expressing disapproval for the expression of PE (punish), ignoring that expression (neglect), or trying to distract the adolescent from that PE (override) also perceived parents as using harsher parenting. More overprotection was also associated with the use of punish and override; being less emotionally warm was also related with the use of punish and neglect. Interestingly, we found a non-significant association between override and emotional warmth, whereas a negative association was expected. This result does not support our hypothesis that override is a maladaptive emotion socialization strategy in all circumstances. In some situations, these reactions may be adequate: for an overjoyed adolescent that was allowed to go out with friends, parents may adaptively ask the adolescent to calm down, in order to make her/him focus on homework. A limitation of this study was that item 10 in override of overjoy (…bought me something I liked) did not contribute to the factor and had to be removed. A possible explanation is that buying a gift is a pleasurable experience, not associated with the downregulation of positive affect. Future work using this instrument could benefit from rewriting this item, but also item 14 as it has also been excluded from the model of neglect because of low factor loadings. This may be the case because this item describes parental maladaptive behavior (…did not pay attention to my overjoy) while the other two items are positive reactions (…took time to focus on me). In the case of the emotion of overjoy, this may also be connected with the low internal consistency of the scale, the only regarding overjoy.

Two additional results regarding NE are worth analyzing. Like in previous research (Klimes-Dougan et al. 2007; O’Neal & Magai, 2005; Silk et al. 2011), the punish scales (NE) emerged with lower levels of internal consistency. Furthermore, we found an unexpected positive relation with emotional warmth and non-significant relations with rejection (while positive with overprotection). This result may be due to the importance of collectivist values for Portuguese parents, even when promoting adolescents autonomy (i.e., interindependence; Prioste, Narciso, Gonçalves, & Pereira, 2015). Parenting in collective cultures (Rudy, Grusec, & Wolfe, 1999) aim to teach youth to learn how to inhibit the expression of their wishes and needs, to self-restraint, and privilege the attendance of others’ needs (e.g., Latin-American and Asian countries). Hence, rearing practices characterized by higher control and imposition of self-restrain, which are parental behaviors included in rejection and overprotection dimensions, might be interpreted positively by Portuguese adolescents because they may not associate it with negative parental intentions or lack of emotional support. This benevolent view of authoritarian practices has been observed in other collective cultures (Rudy et al. 1999).

Although magnifying NE was associated with more overprotection and rejection (only anger), we also found an unexpected positive relation with emotional warmth. Portuguese adolescents may interpret these reactions as twofold: as disturbing (when parents’ negative feelings preclude them from offering support to the adolescent) or as a sign of empathy (as parents are mirroring what adolescents are feeling). Others have already hypothesized magnify to have a double meaning. To be a supportive/empathetic response even for anger, as it may be perceived by youth as parents joining them towards a common cause or as a negative parental response as youth may be the target of their parents’ anger (Klimes-Dougan et al. 2014). Since this is the first work with a Portuguese sample, our explanations about punish and magnify remain tentative.

Other limitations may be pointed out to our investigation. Only two adolescents were used to test the semantic validity of ESS when several groups of participants with at least three members are recommended (Pasquali, 2010). Although adolescents with cognitive impairment were excluded from the sample, previous semantic validity testing could have been reinforced. Also, we did not control for the impact of clinical significant symptoms (e.g., depressive symptoms) in the results. It is possible that a prevalent percentage of clinically significant symptoms (e.g., depressive symptoms) in the sample may distort the way these adolescents interpret their behaviors and their parent’s behaviors and, consequently, the results. If this is proven to be in future studies, researchers should consider establishing clinical symptoms as exclusion criterion for participants’ selection.

## Conclusions

In summary, this investigation provided a methodological contribution to the field, by producing a ESS assessing a PE (overjoy): (i) some evidence of validity (factorial structure and convergent validity) and reliability for the Portuguese version and (ii) an English translation available for future studies. Research should, nonetheless, pursue the refinement of the punish scales and meaning of magnify for NE, and of override for the overjoy scale. Also, pursuing additional validity evidence is important. In particular, as positive emotions have been associated with resilience and flexibility (Fredrickson, 2013; Gruber et al. 2014), it would be relevant to test the relation of the emotion socialization scales of overjoy with these constructs. Moreover, our findings show that parental behaviors considered by many cultures as negative (e.g., punish: disapproval for the emotion expressed by the adolescent) may be perceived by Portuguese adolescents as positive (e.g., positive association with emotional warmth). This calls attention to the relevance of cross-cultural research, as it unveils the idiosyncratic nature of many perceptions and beliefs, highlighting the distinction (Behling & Law, 2000) between semantic (i.e., relative to the phrasing and content of the instrument items) and normative equivalence (i.e., relative to the conformity between the instrument and the cultural rules of the target culture). In the case of our work, although the items are semantically perceived as identical (e.g., items in the punish scale are decoded in both languages as expressing disapproval for the expression of PE), they carry different cultural significance.

From a theoretical standpoint, this investigation also goes further showing that variations on the way mothers react to PE may also be an indicator of the quality of the mother-adolescent relation. Others have already stated that supportive responses to NE (i.e., reward) may be ‘nestled in the web of positive parenting’ (Katz et al. 1999, p. 142). We showed that reactions to youth PE may also matter, adding to recent investigations emphasizing the importance of PE for adaptive and maladaptive development (Gruber et al. 2014). This result may even be more relevant because we studied adolescents and their perception. Although adolescents strive for independence and turn to peers for support and fun, they seem to value parents joining them in sharing positive emotional moments and dislike parental reactions that reject and buffer those emotional experiences.

## Additional files

Additional file 1: Asymmetry and Kurtosis for Individual Items of ESS by Emotion Socialization Strategy and Emotion (*N* = 418). (DOCX 20 kb)

## Authors’ information

ECM is a Lecturer of Psychology at Maia University Institute and a researcher of the Center for Psychology at the University of Porto, Portugal. She conducts investigations on developmental psychopathology (positive emotion, emotion regulation, executive functions, and social cognition) and on group prevention programs featuring preschoolers and adolescents.

LM is a Lecturer of Psychology, a cognitive-behavioral psychotherapist, and the Head of the Department of Social Sciences and Behavior at the Maia University Institute, Portugal. Her research focuses on studying the efficacy of psychological interventions in infancy and adolescence, and the psychotherapy process and outcomes with depressed adults.

FF-S is a lecturer at the Faculty of Psychology and Education Sciences of the University of Porto. His research in Social and Affective Neuroscience at the Laboratory of Neuropsychophysiology (U. Porto) is focused on the perception of emotion across development and the influence of emotion on social cognition.
