# Supplementary material for: New version of the emotion socialization scale with the positive emotion of overjoy: initial validation evidence with Portuguese adolescents
Source: Psicol Reflex Crit. 2018 Apr 3;31:9. doi: 10.1186/s41155-018-0090-3 (PMC6967315; doi:10.1186/s41155-018-0090-3)
Supplement: Supplementary file 1 — Asymmetry and Kurtosis for Individual Items of ESS by Emotion Socialization Strategy and Emotion (N = 418). (DOCX 20 kb) [file 41155_2018_90_MOESM1_ESM.docx]

| *Table 5*  *Asymmetry and Kurtosis for Individual Items of ESS by Emotion Socialization Strategy and Emotion (N = 418)* | | | | | | | | | | |
| --- | --- | --- | --- | --- | --- | --- | --- | --- | --- | --- |
|  |  |  | Sad | | Angry | | Fear | | Overjoy | |
| Emotion socialization strategy | Item |  | Skew | Kurt | Skew | Kurt | Skew | Kurt | Skew | Kurt |
| Reward | 3 |  | -.884 | -.540 | -.279 | -.909 | -.463 | -.869 | -.241 | -.889 |
|  | 6 |  | -.620 | -.699 | -.502 | -.736 | -.540 | -.783 | -.584 | -.531 |
|  | 15 |  | -.776 | -.587 | -.358 | -.977 | -.516 | -.939 | -.570 | -.577 |
| Neglect | 1 |  | -.290 | -.948 | -.085 | -.865 | .034 | -1.014 | -.483 | -.696 |
|  | 12 |  | -389 | -.881 | -.093 | -.995 | -.188 | -1.010 | .086 | -.963 |
|  | 14 |  | 1.323 | .902 | 1.097 | .415 | 1.182 | .513 | .978 | .054 |
| Override | 7 |  | -.253 | -.840 | -.050 | -848 | -.256 | -.904 | .831 | -155 |
|  | 10 |  | .374 | -.764 | .642 | -.445 | .732 | -.321 | .477 | -.804 |
|  | 11 |  | -.818 | -322 | -.349 | -.990 | -.566 | -.722 | .719 | -373 |
| Punish | 2 |  | -.617 | -.709 | -.339 | -.773 | -.355 | -.939 | 1.79 | 2.034 |
|  | 5 |  | .978 | -.008 | .756 | -.352 | 1.151 | .524 | 1.164 | .193 |
|  | 9 |  | .462 | -.935 | .465 | -.843 | .695 | -.655 | 1.701 | 1.704 |
| Magnify | 4 |  | .302 | -860 | .918 | .168 | 1.062 | .269 | -.645 | -.524 |
|  | 8 |  | .520 | -.665 | .864 | -.004 | 1.225 | .927 | -.299 | -.965 |
|  | 13 |  | .474 | -.751 | .773 | -.151 | .978 | .220 | .256 | -.949 |
